# Visible Foliar Injury and Physiological Responses to Ozone in Italian Provenances of *Fraxinus excelsior* and *F. ornus*

**DOI:** 10.1100/tsw.2007.10

**Published:** 2007-03-21

**Authors:** Nicla Contran, Elena Paoletti

**Affiliations:** ^1^ Department of Environmental Sciences, University of Milano-Bicocca, Piazza della Scienza 1, Milan, Italy; ^2^ IPP-CNR, Via Madonna del Piano 10, I-50019 Sesto Fiorentino, Florence, Italy

**Keywords:** ash trees, foliar injury, fluorescence, gas exchange, ozone

## Abstract

We compared leaf visible injury and physiological responses (gas exchange and chlorophyll a fluorescence) to high O_3_ exposure (150 nmol mol^–1^ h, 8 h day^–1^, 35–40 days) of two woody species of the same genus with different ecological features: the mesophilic green ash (*Fraxinus excelsior*) and the xerotolerant manna ash (*F. ornus*). We also studied how provenances from northern (Piedmont) and central (Tuscany) Italy, within the two species, responded to O_3_ exposure. Onset and extent of visible foliar injury suggested that *F. excelsior* was more O_3_ sensitive than *F. ornus*. The higher stomatal conductance in *F. ornus* than in *F. excelsior* suggested a larger potential O_3_ uptake, in disagreement to lower visible foliar injury. The higher carbon assimilation in *F. ornus* suggested a higher potential of O_3_ detoxification and/or repair. Contrasting geographical variations of ash sensitivity to O_3_ were recorded, as Piedmont provenances reduced gas exchange less than Tuscan provenances in *F. excelsior* and more in *F. ornus*. Visible injury was earlier and more severe in *F. excelsior* from Piedmont than from Tuscany, while the provenance did not affect visible injury onset and extent in *F. ornus*.

### INTRODUCTION

Tropospheric ozone ($O_3$) is regarded as one of the most widespread air pollutants[1]. Current $O_3$ levels in Europe are potentially high enough to adversely affect forests[1]. Tree species exhibit a wide range of sensitivity, even at the intraspecific level[2,3,4,5,6,7]. Ozone exposure can modify important physiological processes, such as photosynthesis and stomatal function[2]. Species-specific chlorotic flecking, necrosis, or bronzing may appear on the upper leaf surface[7]. Degree and type of invisible and visible $O_3$ foliar injuries depend on several plant factors: stomatal conductance, leaf morphological features, apoplastic detoxification, and the response that plants are able to activate[8,9]. These factors are strongly dependent on genotype and on the ecological strategies that plants adopt to avoid or tolerate $O_3$ stress.

A first objective of this investigation was to perform a preliminary screening of leaf visible injury and physiological responses (leaf gas exchange, chlorophyll *a* fluorescence) to high $O_3$ concentrations in seedlings of two woody species of the same genus with different ecological features: the mesophilic green ash (*Fraxinus excelsior* L.) and the xerotolerant manna ash (*F. ornus* L.). *F. excelsior* is considered an $O_3$-sensitive plant and activates a hypersensitive response to $O_3$[10,11]. $O_3$ responses of *F. ornus* have never been investigated. We hypothesize that this species is oxidative-stress tolerant, since it is drought tolerant[6] and thus should have a good pool of constitutive enzymatic and nonenzymatic antioxidants or should be able to increase antioxidant defense on oxidative stress[12].

A second objective was to study preliminarily whether diverse provenances exhibited different responses to high $O_3$ concentrations in terms of leaf visible injury and physiological responses (leaf gas exchanges, chlorophyll *a* fluorescence). We investigated provenances from northern and central Italy within the two species. The aim was to test the hypothesis of a relationship between plants' sensitivity to $O_3$ and a geographical gradient, thought as an ecological gradient. As a result of the adaptation to the environment, plants of different provenances may exhibit different physiological features, which in turn differently influence $O_3$ sensitivity at the intraspecific level[3,13].

### MATERIAL AND METHODS

#### Plant Material and $O_3$ Exposure

Two-year-old potted uniform-size seedlings of *F. excelsior* and *F. ornus* were collected from forest nurseries of Piedmont (northern Italy) and Tuscany (central Italy). Pots were 20 cm in diameter and filled with two-thirds potting medium and one-third vermiculite. The seed sources were of local origin: three provenances from Piedmont and one from Tuscany for *F. excelsior*, and one provenance from Piedmont and one from Tuscany for *F. ornus*. Three months before $O_3$ exposure, 20 seedlings from each provenance were moved to a greenhouse. Seedlings were fertilized with Osmocote and watered to field capacity once a week. One week before $O_3$ exposure, six plants from each group were randomly selected (three as controls and three for $O_3$ exposure per provenance, for a total of 36 seedlings) and allowed to acclimatize in a growth chamber, ventilated with charcoal-filtered air (two air changes per minute) at 20 ± 1°C, 85 ± 5% RH, and 500 $\mu$mol $m^{-2}$ $sec^{-1}$ photon flux density (PPFD) at plant height during a 14-h photoperiod. In June 2004, the seedlings were moved to a charcoal-filtered chamber and an $O_3$-enriched chamber, where their position was rotated once a week. Both chambers were located in the same growth chamber. The environmental conditions were as above. $O_3$ was generated by a Model 500 $O_3$ generator (Fisher, Zurich, Switzerland) supplied with pure $O_2$. Its concentration was continuously monitored with a PC-controlled photometric analyzer (Monitor Labs mod. 8810, San Diego, CA). The exposure regime was a square wave of 150 nmol $mol^{-1}$, from 10:00–18:00 (GMT), for 40 days. The AOT40 accumulated exposure over a threshold of 40 nmol $mol^{-1}$[14] yielded 35.2 $\mu$mol $mol^{-1}$ h.

#### Visible Ozone Injury

Leaves were surveyed daily to detect the onset of visible $O_3$ injury. At the end of $O_3$ exposure, visible injury was assessed by: (1) counting the number of injured seedlings, expressed as the percentage of injured seedlings of all the seedlings present; (2) counting the number of leaflets showing visible injury, expressed as the percentage of injured leaflets of all leaflets present (%I.L.); and (3) visually assessing the percent surface injury (according to the guide in Innes et al.[15]), expressed as the percentage of injured leaflet surface per symptomatic leaflet surface (%L.A.). The position of each symptomatic leaf and leaflet was recorded, with the apical one as position 1.

#### Gas Exchange and Chlorophyll *a* Fluorescence

Measurements were carried out at 7-day intervals on the adaxial surface of subapical leaflets of two fully expanded leaves per plant, on three plants per provenance and per exposure. The measured leaves were free of any symptom. Steady-state measurements of light-saturated photosynthesis ($P_{net}$) and stomatal conductance to water vapor ($G_w$) were made using $O_3$-free air by an infrared gas analyzer (CIRAS-1 PP-System, Herts, U.K.) equipped with a Parkinson leaf cuvette, which controlled leaf temperature (26 ± 1°C), leaf-to-air vapor pressure deficit (1 ± 0.2 kPa), light (1300 ± 20 µmol $m^{-2}$ $sec^{-1}$ PPFD), and $CO_2$ concentration (360 ± 10 µmol $mol^{-1}$). Chlorophyll *a* transient fluorescence was measured *in vivo* with a direct fluorometer (Handy PEA, Hansatech Instr., Kings Lynn, U.K.). Before measurement, leaves were dark adapted for 40 min with leaf clips. The rising transient was induced by saturating red-actinic light (1300 µmol $m^{-2}$ $sec^{-1}$, peak at 650 nm, duration 1 sec). Data acquisition was recorded for 1 sec, starting from 10 µsec after the onset of illumination. The values of Fo, i.e., ground fluorescence yield in the dark-adapted state (when all reaction centers of PSII are considered open) and Fm, i.e., the maximal fluorescence yield in the dark (when all reaction centers of PSII are considered closed), were collected. Maximum quantum yield for primary photochemistry (Fv/Fm) was calculated as (Fm-Fo)/Fm[16].

After 3 weeks of exposure, $A/C_i$ curves and Performance Index were measured. A/Ci curves were obtained by changing $CO_2$ concentration entering the cuvette ($C_a$) from 50 to 1000 µmol $mol^{-1}$, in light-saturated condition (1300 ± 20 µmol $m^{-2}$ $sec^{-1}$ PPFD), at constant leaf temperature (26 ± 1°C) and leaf-to-air vapor pressure deficit (1 ± 0.2 kPa). Steady-state $CO_2$ assimilation was first measured by setting the reference $CO_2$ concentrations near ambient (400 µmol $mol^{-1}$) and then at 300, 200, 100, 50, 400, 500, 700, and 1000 µmol $mol^{-1}$[17]. Maximum RuBP-saturated rate of carboxylation ($V_{cmax}$), mitochondrial respiration rate in the light ($R_{day}$), and maximum rate of electron transport ($J_{max}$) were estimated with an iterative procedure according to Farquhar et al.[18] and Harley et al.[19]. Performance Index ($P.I._{abs}$), an indicator for the photosynthetic status of the leaf, was acquired by the JIP-test with Biolyzer 3.06 software (by Ronald Maldonado-Rodriguez, Biogenetics Laboratory, Geneva, Switzerland)[20].

### Data and Statistical Analyses

Data were checked for normal distribution (Shapiro-Wilk W test) and homogeneity of variance (Levene's test). Percents were arcsine-square root transformed prior to analysis. A preliminary ANOVA did not show significant differences among the three Piedmont provenances of *F. excelsior*. Therefore, they were treated as only one statistical group. For data collected weekly, effects of $O_3$ exposure, species, and provenance were tested with a repeated-measure ANOVA, with time as repeated-measure factor. For data collected after 3 weeks of exposure, effects of $O_3$ exposure, species, and provenance were tested with a three-way ANOVA. A t-test was applied to compare the effects of $O_3$ exposure at each date of measurement. Tests of significance were made at a 95% confidence level. Analyses were processed using STATISTICA 6.0 Package for Windows (StatSoft 2001, Tulsa, OK).

## RESULTS

Both species displayed interveinal reddish stipples on the adaxial leaf surface. Visible injury was earlier and more severe in *F. excelsior* than in *F. ornus* (Table 1). In *F. excelsior*, the Piedmont provenances appeared to be more $O_3$ sensitive than the Tuscan provenances, in terms of visible injury, while the provenance did not affect visible injury onset and extent in *F. ornus*. However, a strong data variability prevented statistical significance. Both for species and provenances, the position of leaf and leaflet did not affect %I.L and %L.A (data not shown).

**TABLE 1**

**Onset and Extent (Percent of Injured Seedlings, of Injured Leaflets [I.L.], and of Injured Surface per Symptomatic Leaflet [L.A.]) of Visible Ozone Injury in Piedmont and Tuscan Provenances of *F. excelsior* and *F. ornus*, Exposed to 150 nmol $O_3$ mol$^{-1}$ (8 h day$^{-1}$, 40 days) and F Values of ANOVA of the Effects of Species (*F. excelsior* and *F. ornus*) and Provenance (Piedmont and Tuscany)**

| | Onset (day of exposure) | Injured seedlings (%) | I.L. (%) | L.A. (%) |
|---|---|---|---|---|
| ***Fraxinus excelsior*** | | | | |
| from Piedmont | 12 | 78 | 54.3 | 9.03 |
| from Tuscany | 21 | 67 | 31.2 | 4.04 |
| ***Fraxinus ornus*** | | | | |
| from Piedmont | 37 | 67 | 8.05 | 1.04 |
| from Tuscany | 38 | 33 | 7.05 | 1.05 |
| Species | - | - | $1.16^{ns}$ | $3.55^{ns}$ |
| Provenance | - | - | $0.44^{ns}$ | $0.14^{ns}$ |
| Species x Provenance | - | - | $0.01^{ns}$ | $0.67^{ns}$ |

ns= $p > 0.05$ (not significant).

$O_3$ exposure significantly decreased $P_{net}$, $G_w$, and Fv/Fm (Table 2). $P_{net}$ and $G_w$ values were higher in *F. ornus* than in *F. excelsior,* and in the seedlings from Tuscany than in those from Piedmont, both in $O_3$-exposed and control samples. In both species, $P_{net}$ declined mostly during the first week of $O_3$ exposure and then it was stable (Fig. 1). $P_{net}$ decline was faster in *F. excelsior* (–59% after 1 week of exposure) than in *F. ornus* (–31% after 1 week and –56% after 2 weeks). While $G_w$ of *F. ornus* showed the same change as $P_{net}$, *F. excelsior* took 3 weeks of $O_3$ exposure to show a significant decrease in $G_w$ of $O_3$-exposed seedlings. In *F. excelsior* control, $G_w$ remained constant while in *F. ornus,* it increased over time. Therefore, $G_w$ decrease in *F. ornus* ($O_3$-exposed vs. control) was higher than in *F. excelsior*. In $O_3$-exposed plants of both species, Fv/Fm decreased substantially in a similar way (4% after 1 week of exposure) and then it kept constant over time in *F. excelsior*, while an increase after 28 days of exposure was observed in *F. ornus* when the efficiency of PSII recovered to control values.

In $O_3$-exposed F. ornus, $P_{net}$ and $G_w$ of Tuscan provenance decreased more slowly than those of Piedmont provenance. In $O_3$-exposed F. excelsior, $P_{net}$ and $G_w$ of Tuscan provenance decreased at a larger extent than those of Piedmont provenances. The effects of $O_3$ exposure on Fv/Fm were observed only in the Piedmont provenances of F. excelsior and in the Tuscan provenance of F. ornus.

$R_{day}$ was higher in F. ornus than in F. excelsior, but did not vary with $O_3$ exposure and provenance (Table 3). On the contrary, $V_{cmax}$, $J_{max}$, and $P.I._{abs}$ showed a significant reduction in $O_3$-exposed seedlings. They were significant lower in Piedmont provenances than in Tuscan provenances of both species. While $V_{cmax}$ and $J_{max}$ showed no differences between the species, $P.I._{abs}$ was significantly higher in *F. ornus* than in *F. excelsior*.

**TABLE 2**
**F Values of Three-Way Repeated ANOVA of the Effects of $O_3$ Exposure (0 and 150 nmol $mol^{-1}$, 8 h $day^{-1}$), Species (*F. excelsior* and *F. ornus*), and Provenance (Piedmont and Tuscany) with Time as Repeated-Measure Factor (0, 7, 14, 21, 28, and 35 Days of Exposure)**

| Effects | d.f. | $P_{net}$ | $G_w$ | Fv/Fm |
|---|---|---|---|---|
| $O_3$ exposure | 1 | 299.2*** | 289.3*** | 139.9*** |
| Species | 1 | 91.18*** | 188.5*** | 34.66*** |
| Provenance | 1 | 127.9*** | 8.11* | 19.56*** |
| Species x Provenance | 1 | 24.75*** | $1.59^{ns}$ | 30.52** |
| $O_3$ exposure x Provenance | 1 | 51.83*** | $3.13^{ns}$ | 8.29** |
| $O_3$ exposure x Species | 1 | $3.21^{ns}$ | 68.78*** | 6.98* |
| $O_3$ exposure x Species x Provenance | 1 | $4.04^{ns}$ | 18.65*** | 22.86*** |
| Time | 5 | 4.27*** | 12.99*** | 10.87*** |
| Time x $O_3$ exposure | 5 | 6.75*** | 15.89*** | 1.11*** |
| Time x Provenance | 5 | 5.98*** | 12.19*** | 4.26*** |
| Time x Species | 5 | 14.41*** | 12.23*** | 13.65*** |
| Time x Species x Provenances | 5 | 13.53*** | 17.01*** | 4.01*** |
| Time x $O_3$ exposure x Provenances | 5 | 6.07*** | 20.73*** | $0.58^{ns}$ |
| Time x $O_3$ exposure x Species | 5 | 4.18** | 23.03*** | 3.67** |
| Time x $O_3$ exposure x Species x Provenances | 5 | 9.89*** | 23.85*** | $1.53^{ns}$ |

d.f. represents the degrees of freedom; *=$p \leq 0.05$, **=$p \leq 0.01$, ***=$p \leq 0.001$, ns = $p > 0.05$ (not significant).

For abbreviations see Fig. 1.

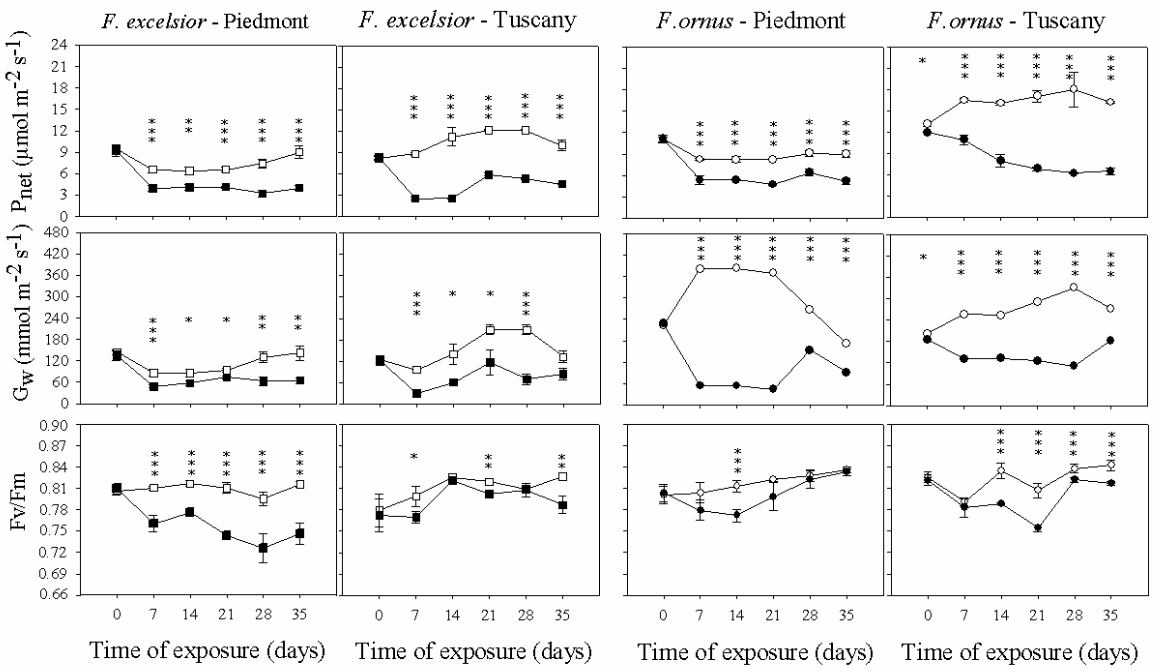

**FIGURE 1**. Effects of ozone exposure (150 nmol $mol^{-1}$, 8 h $day^{-1}$, 35 days) on net photosynthesis ($P_{net}$), stomatal conductance to water vapor ($G_w$), and maximum quantum yield for primary photochemistry (Fv/Fm) in Piedmont and Tuscan provenances of *F. excelsior* and *F. ornus*. Values represent means ±S.E. Symbols represent the results of t-test of $O_3$ exposure. *, $p \leq 0.05$; **, $p \leq 0.01$; ***, $p \leq 0.001$; ns (not significant), $p > 0.05$; □, *F. excelsior* control; ■, *F. excelsior* $O_3$ exposed; ○, *F. ornus* control; ●, *F. ornus* $O_3$ exposed.

**TABLE 3**
**Mean Values (±S.E.) of Mitochondrial Respiration Rate in the Light ($R_{day}$[μmol $m^{-2}$ $sec^{-1}$]), Maximum RuBP-Saturated Rate of Carboxylation ($V_{cmax}$[μmol $m^{-2}$ $sec^{-1}$]), Maximum Rate of Electron Transport ($J_{max}$[μmol $m^{-2}$ $sec^{-1}$]), and Performance Index ($P.I._{abs}$) and F Values of Three-Way ANOVA of the Effects of $O_3$ Exposure (0 and 150 nmol $mol^{-1}$ h, 8 h $day^{-1}$, 21 days), Species (*F. excelsior* and *F. ornus*) and Provenances (Piedmont and Tuscany)**

| | | | $R_{day}$ | $V_{cmax}$ | $J_{max}$ | $P.I._{ABS}$ |
|---|---|---|---|---|---|---|
| ***F. excelsior*** | | | | | | |
| | Piedmont | Control | 2.56 (±0.56) | 93.93 (±9.41) | 128.03 (±16.81) | 58.19 (±7.01) |
| | | Treated | 1.90 (±0.23) | 41.37(±5.03) | 60.90 (±6.72) | 35.58 (±4.42) |
| | Tuscany | Control | 1.56 (±0.68) | 124.00 (±7.49) | 171.61 (±14.51) | 96.21 (±4.31) |
| | | Treated | 3.16 (±0.85) | 90.82 (±10.00) | 111.12 (±11.19) | 64.34 (±6.89) |
| ***F. ornus*** | | | | | | |
| | Piedmont | Control | 4.746 (±1.21) | 88.26 (±17.34) | 111.53 (±23.69) | 79.42 (±8.03) |
| | | Treated | 3.69 (±1.55) | 63.59 (±10.14) | 88.05 (±15.76) | 52.12 (±8.42) |
| | Tuscany | Control | 3.56 (±2.11) | 125.91 (±1.69) | 167.00 (±18.18) | 110.28 (±3.74) |
| | | Treated | 4.29 (±1.03) | 61.83 (±16.67) | 79.46 (±17.26) | 71.59 (±4.80) |
| | **Effects** | **d.f.** | | | | |
| | $O_3$ exposure | 1 | $0.034^{ns}$ | 31.90*** | 27.08*** | 36.75*** |
| | Species | 1 | 4.570* | $0.117^{ns}$ | $0.312^{ns}$ | 8.799* |
| | Provenance | 1 | $0.009^{ns}$ | 13.93** | 9.405** | 34.604*** |
| | $O_3$ exposure x Species | 1 | $1.144^{ns}$ | $0.009^{ns}$ | $0.131^{ns}$ | $0.329^{ns}$ |
| | $O_3$ exposure x Provenance | 1 | $1.146^{ns}$ | $0.317^{ns}$ | $1.566^{ns}$ | $1.068^{ns}$ |
| | Species x Provenance | 1 | $0.066^{ns}$ | $1.987^{ns}$ | $1.045^{ns}$ | $0.675^{ns}$ |
| | $O_3$ exposure x Species x Provenance | 1 | $0.019^{ns}$ | $3.624^{ns}$ | $2.378^{ns}$ | $0.012^{ns}$ |

d.f. represents the degrees of freedom; *=p ≤ 0.05, **=p ≤ 0.01, ***=p ≤ 0.001, ns= p > 0.05 (not significant).

### DISCUSSION

Onset and extent of visible foliar injury as well as faster decline in photosynthesis suggested that *F. excelsior* was more sensitive to $O_3$ than *F. ornus*. In both species, gas exchange and chlorophyll *a* fluorescence measurements showed that $O_3$ affected photosynthetic performances. After 1 week of exposure, $O_3$ significantly reduced $P_{net}$ and $G_w$. Even if the experiment lasted only 40 days, the plants adapted to $O_3$ since gas exchange kept constant during the following weeks. The decrease in carbon fixation was associated with a reduction in $G_w$ as well as in the quantity of active Rubisco ($V_{cmax}$) and the capacity for whole-chain electron transport ($J_{max}$). The decrease of $P.I._{abs}$ confirmed that the energy transduction process around PSII lost performance. Thus, in these ash species, $O_3$ effects on photosynthesis resulted from effects on stomata and on the photosynthetic apparatus[8].

Avoidance by stomatal regulation, limiting the access of $O_3$ to sensitive targets, is the first mechanism of plant $O_3$ sensitivity[21,22]. Accordingly, the more $O_3$-tolerant species (*F. ornus*) had the stronger reduction of $G_w$ ($O_3$-exposed seedlings vs. controls). However, $G_w$ values were lower in *F. excelsior* than in *F. ornus*. Probably, in a controlled environment without water stress, the xerotolerant *F. ornus* was allowed to maximize its stomatal capacity. The values of $G_w$ suggest that the potential $O_3$ uptake in *F. excelsior* plants was lower than in *F. ornus*, even if the former species was more sensitive to $O_3$. Species-specific $G_w$ is not necessarily correlated with $O_3$ sensitivity based on the severity of foliar injury[23]. Two other factors control plant $O_3$ sensitivity: (1) plant resources available for repair of damaged tissues and (2) plant enzymatic and nonenzymatic antioxidant levels[8,9]. Therefore, in condition of stomatal conductance equality, net photosynthesis has been suggested as a better indicator of plant sensitivity to $O_3$ because the availability of photosynthate is particularly important in antioxidant defense and repair mechanisms[23,24,25]. This hypothesis implies that high rates of net photosynthesis may balance $O_3$ uptake and reduce foliar injury[26]. In agreement to this view, *F. ornus* had higher $P_{net}$ and $R_{day}$ values. The repair capacity of *F. ornus* is supported by the recovery of efficiency of PSII (Fv/Fm) after 4 weeks of $O_3$ exposure. Plants adapted to high oxidative stress levels, like the xerotolerant *F. ornus,* may be less sensitive to $O_3$ exposure[27] because of a good pool of constitutive enzymatic and nonenzymatic antioxidant levels and/or the ability to increase antioxidant defenses.

Based on visible foliar injury, no significant difference in $O_3$ sensitivity was observed between the provenances. Based on gas exchange, the provenances of the two species differed in their response to $O_3$ in that the Piedmont provenances reduced gas exchange less than the Tuscan provenances in *F. excelsior* and more in *F. ornus*. As a correlation between provenances and $O_3$ sensitivity has been demonstrated in other studies[3,13], it is possible that our provenances were not too far away to show different $O_3$ responses, and that the results were affected by the small replication and short term of the experiment.

### ACKNOWLEDGMENTS

The study was funded by the Regione Piemonte (project FORMEDOZON, Interreg III B). The authors wish to thank Francesco Tagliaferro for providing the plant material from Piedmont, Giacomo Lorenzini and Cristina Nali for performing the ozone exposure, and Francesco Fava, Raffaella Cerana, and Gianni Della Rocca for their helpful comments and practical support.
